# Predicting Treatment Outcomes in Guided Internet-Delivered Therapy for Anxiety Disorders—The Role of Treatment Self-Efficacy

**DOI:** 10.3389/fpsyg.2021.712421

**Published:** 2021-10-21

**Authors:** Adrian Schønning, Tine Nordgreen

**Affiliations:** ^1^Faculty of Psychology, University of Bergen, Bergen, Norway; ^2^Division of Psychiatry, Haukeland University Hospital, Bergen, Norway

**Keywords:** self-efficacy, guided internet-delivered therapy, panic disorder, social anxiety disorder, anxiety disorders, symptom reduction, dropout

## Abstract

**Aim:** Guided Internet-delivered therapy has shown to be an effective treatment format for anxiety disorders. However, not all patients experience improvement, and although predictors of treatment outcome have been identified, few are consistent over time and across studies. The current study aimed to examine whether treatment self-efficacy (self-efficacy regarding the mastery of obstacles during treatment) in guided Internet-delivered therapy for anxiety disorders in adults could be a predictor of lower dropout rates and greater symptom reduction.

**Method:** The analyzed data comes from an open effectiveness study including 575 patients receiving guided Internet-delivered therapy for panic disorder or social anxiety disorder. Treatment self-efficacy was measured at pre-treatment. Symptom reduction was measured at 10 measurement points, including a 6-month follow-up. A mixed linear model was applied in the analysis.

**Results:** The results showed that high treatment self-efficacy was a predictor of both lower dropout rates and greater symptom reduction. Significant interaction effects between time and treatment self-efficacy were found for several of the nine modules that constitutes the treatment program, suggesting that treatment self-efficacy could be a moderator of symptom reduction. Three of nine modules in the panic disorder treatment and six of nine in the social anxiety disorder treatment showed significant interaction effects.

**Conclusion:** The results suggest that measuring treatment self-efficacy may be a valuable tool to identify patients at risk of dropping out, and that treatment self-efficacy could be a predictor and moderator of symptom reduction in guided Internet-delivered therapy. The implications of the results are discussed.

## Introduction

Anxiety disorders are chronic, disabling disorders with a worldwide presence. Anxiety disorders were the sixth leading cause of disability across countries, measured through years of life lived with disability (Baxter et al., 2014). Research indicate that anxiety disorders are among the most prevalent mental disorders with epidemiological studies from USA and Europe showing that anxiety disorders has a lifetime prevalence of 14.5–33.7% in adults (Bandelow and Michaelis, 2015). In terms of socioeconomic costs, it has been estimated that anxiety disorders in 2010 cost 74.4 billion (Olesen et al., 2012). Panic disorder (PD) and social anxiety disorder (SAD) are both among the most prevalent anxiety disorders with lifetime prevalence of, respectively, 1.6–5.2% and 2.8–13% (Bandelow and Michaelis, 2015). Both disorders are associated with reduced social function, increased risk of dropout from school and work, and increased risk of comorbid illnesses such as major depressive disorder and substance abuse (Wittchen, 1988; Klerman et al., 1991; Furmark, 2002; Goodwin et al., 2005; Stein and Stein, 2008).

Cognitive Behavioral Therapy (CBT) is proven an effective treatment for PD and SAD, with support from both efficacy- and effectiveness trials (Stewart and Chambless, 2009; Cuijpers et al., 2016). Although effective treatment options for PD and SAD exist, few of those afflicted receive treatment in routine care—a phenomenon referred to as the treatment gap (Kohn et al., 2004; Shafran et al., 2009). A review of 37 epidemiological studies suggest that the treatment gap for PD is 55.9%, i.e., 55.9% of PD patients remain untreated (Kohn et al., 2004). This calls for ways in which more patients can receive treatment for their disorder. A potential way of reducing the treatment gap is by delivering the interventions *via* the Internet (van Ballegooijen et al., 2016).

The efficacy of guided Internet-delivered therapy is well-established in numerous of efficacy trials, measuring effect in controlled research settings (Andersson, 2016), and it seems that these results can be transferred to clinical settings (Andersson and Hedman, 2013; El Alaoui et al., 2015a; Titov et al., 2016; Andersson et al., 2018). Systematic reviews of Randomized Controlled Trials (RCTs) of Internet-based Cognitive Behavioral Therapy (ICBT) has found moderate to strong effects at posttreatment, which are similar effects as those found for face-to-face Cognitive Behavioral Therapy (CBT) (Cuijpers et al., 2007; Spek et al., 2007). A Cochrane review examining Guided ICBT for anxiety disorders in adults concluded that the evidence suggests that there might be no significant differences in efficacy between guided ICBT and face-to-face CBT in reducing anxiety (Olthuis et al., 2015). The essential question in the field of guided Internet-delivered therapy is not whether it is effective or not, but rather for whom it works and what characteristics might be associated with successful treatment, and which mechanisms guided Internet-delivered therapy operates through (Andersson et al., 2008, 2009; Andersson and Hedman, 2013; Andersson, 2016). Unfortunately, there are challenges related to Internet-delivered interventions, such as high dropout rates and patients not experiencing sufficient treatment effects. Many patients who receive psychological treatment (both ICBT and CBT) do not achieve clinically significant improvement (Taylor et al., 2012; Boettcher et al., 2013). Increased knowledge of predictors of treatment outcomes is especially important for effectiveness trials, as research here is scarcer and will likely increase the possibility to discover more robust predictors of treatment outcome in clinical practice, as compared to efficacy trials (Andersson and Hedman, 2013). Treatment outcomes can be investigated both in a patient's changes in symptoms throughout the treatment and in dropout from treatment (Edmonds et al., 2018). The knowledge of predictors of treatment outcomes in guided Internet-delivered therapy can be used to offer patients the treatment they are most likely to respond to, and to adapt the treatment more effectively to patients who are less likely to respond.

Previous research on predictors of symptom reduction has resulted in somewhat inconsistent results (Keijsers et al., 1994; O'Rourke et al., 1996; McCusker et al., 2000; Carlbring et al., 2001, 2006; Hedman et al., 2012; El Alaoui et al., 2015b). Intensity of baseline symptoms seems to be one of the most stable predictors of symptom reduction, and contrary to what one might expect, higher levels of baseline symptoms are not necessarily associated with negative treatment outcomes (Nordgreen et al., 2012; El Alaoui et al., 2015b; Hadjistavropoulos et al., 2015; Edmonds et al., 2018). Some studies suggest that the magnitude of improvement during and after treatment is not affected by the pre-treatment symptom levels (Eskildsen et al., 2010; Nordgreen et al., 2012). Other studies report that higher pre-treatment symptom levels predict improvement in a wide range of mental disorders, including SAD (Nordgreen et al., 2012; Andersson et al., 2014a; Hadjistavropoulos et al., 2015; Edmonds et al., 2018). Contrary to this, others report that higher pre-treatment symptom levels predict poorer treatment outcomes. For example, some studies have found that severity of panic symptoms in PD was negatively associated with treatment outcomes (Haby et al., 2006; Dow et al., 2007; El Alaoui et al., 2013). Although evidence for the intensity of baseline symptoms as a predictor of treatment outcome exist, the results are still somewhat inconsistent. Therapeutic alliance has been examined as a potential predictor of treatment outcome, as guided treatment typically yields better results than unguided treatment (Lewis et al., 2012). Unfortunately therapeutic alliance rarely predicts outcome (Andersson and Hedman, 2013). Andersson et al. (2014b) concludes that the research on predictors of outcome in ICBT for SAD is limited, but that traditional predictors such as therapeutic alliance most likely are not of significant importance for SAD. For example, Andersson et al. (2012) found that although the therapeutic alliance in guided ICBT for depression, generalized anxiety disorder and SAD was rated high and in line with face-to-face studies, the correlation between Working Alliance Inventory (WAI) and symptomatic change were small and not statistically significant. Although some potential predictors of treatment outcome have been discovered, there is yet no well-established knowledge regarding predictors of symptom reduction in guided Internet-delivered therapy. Overall, it remains unclear which factors predict symptom reduction, despite the growing literature (Andersson et al., 2008; Andersson, 2016).

Although guided Internet-delivered therapy has proven itself as an effective treatment option, high dropout rates remain a problem (Eysenbach, 2002; Wangberg et al., 2008; Donkin et al., 2011; Beatty and Binnion, 2016). Dropout is defined and used in various ways, but in general dropout can be defined as leaving treatment before its completion (Davis et al., 2006). Dropout can occur at any point throughout the treatment, and various types of dropout are often classified thereafter (Eysenbach, 2005). Even though dropout in guided Internet-delivered therapy is a problem, high dropout rates constitute a problem in all therapy, also in face-to-face CBT. Some research indicates that there might be no differences in dropout rates between guided ICBT and face-to-face CBT. A meta-analysis summarizing data from 24 RCTs found that patients with depression in face-to-face CBT complete an entire intervention more often than patients with depression in guided ICBT (face-to-face: 84.7%, ICBT: 65.1%) (van Ballegooijen et al., 2014). However, the same study found that non-completers in guided ICBT completed more of the interventions than non-completers in face-to-face CBT (42.1% of the interventions vs. 24.5%) and the authors concludes that adherence to guided ICBT could be equal to adherence in face-to-face CBT. The completion of modules in Internet-delivered interventions seem to predict symptom reduction across mental disorders and for SAD specifically, which makes the reduction of dropout a central goal in Internet-delivered interventions (Donkin et al., 2011; El Alaoui et al., 2015b; Hedman et al., 2016). Eysenbach (2005) claims that low adherence and dropout are main barriers to the effectiveness of Internet-delivered interventions. With this in mind, it is argued that dropout needs to be examined not only as an predictor of outcome, but also as an outcome in itself (Chen et al., 2020).

As for predictors of symptom reduction, the results from research on predictors of dropout in guided Internet-delivered therapy for SAD and PD are inconsistent, and to the best of our knowledge no sound predictors of dropout have been discovered. Melville et al. (2010) identified three categories of predictors of dropout in Internet-delivered psychological treatment, namely: sociodemographic and contextual variables (i.e., age, gender, relationship status), psychological problems (severity of target disorder) and treatment-related variables (i.e., treatment credibility and Internet/computer experience). However, many of the predictors were found not to be significant, and many of the predictors had conflicting results, i.e., being significant in some studies and not in others. Melville et al. (2010) concludes that although variables associated with dropout have been explored, evidence on predictors of dropout is limited. Other reviews report similar findings (Christensen et al., 2009). More recent research has found that patients with anxiety and/or depression who fully completed guided ICBT programs were older and had lower psychological distress at intake than those who only partially completed the programs (Edmonds et al., 2018). A systematic review found that, for a wide range of mental disorders, females, high treatment credibility, having enough time to follow the treatment, having guidance, and personalized intervention content predicted higher adherence in unguided Internet-delivered interventions (Beatty and Binnion, 2016). The same review also found that age, baseline symptom severity and control group allocation showed mixed results, and that most variables included in the review did not predict adherence. El Alaoui et al. (2015b) investigated predictors of adherence in guided ICBT for SAD in routine psychiatric care and found that the strongest predictor of adherence was treatment credibility, and that Attention-Deficit/Hyperactivity Disorder (ADHD)-like symptoms, male gender and family history of depression predicted lower adherence. Guided, compared to unguided Internet-delivered therapy, seem to result in lower dropout rates (Nordgreen et al., 2012). Even though some potential predictors of dropout have been discovered, there is a need for identifying more robust predictors.

Self-efficacy is considered a transdiagnostic mechanism of change for psychological treatments and is believed to play a central role in the development and treatment of anxiety (Bandura, 1977, 1997). Self-efficacy seems to play a central role in understanding behavioral change in PD and SAD (Gallagher et al., 2013; Iancu et al., 2015). Self-efficacy is contingent on specific contexts and behaviors and constitutes a broad and nuanced construct that varies from situation to situation for the same person. Due to its wideness it is claimed that self-efficacy should be assessed at a domain- or task-specific level because such increased specificity enhances validity and predictive properties (Pajares and Schunk, 2001; Salanova et al., 2002). The domain-specific self-efficacy utilized in this study is treatment self-efficacy, i.e., self-efficacy regarding the mastery of obstacles during use of the guided Internet-delivered therapy.

Results from CBT studies for PD suggest that self-efficacy might be a predictor of symptom reduction in PD. Self-efficacy for managing PD symptoms has been shown to predict PD symptom severity (Telch et al., 1989; Richards et al., 2002). A systematic review claim that, although several methodological issues exist, the literature provide some support for panic self-efficacy (the perceived ability to cope with panic attacks) as a mediator of outcome in CBT for PD (Fentz et al., 2014). For example, Gallagher et al. (2013) found that self-efficacy for coping with PD symptoms mediated symptomatic changes in CBT for PD as it increased throughout the treatment, predicted changes in PD symptoms and preceded symptomatic changes. CBT for PD is believed to foster self-efficacy through experiences of mastery in which beliefs about being able to effectively cope with PD symptoms are developed, in combination with the therapist's persuasion, challenging of the perceived dangers of panic symptoms and encouragement of the patient's ability to cope with the symptoms (Casey et al., 2004). However, it should be emphasized that the mentioned studies investigated the role of panic self-efficacy, and not treatment self-efficacy.

Research comparing SAD patients with healthy controls suggest that low general self-efficacy correlates with high symptom severity in SAD patients (Iancu et al., 2015). It is argued that self-efficacy plays a vital role in explaining behavioral change in SAD. Goldin et al. (2012) found that CBT, compared to a waiting list, led to greater increases in cognitive reappraisal self-efficacy (i.e., the belief that one successfully can implement cognitive reappraisals to regulate emotions) and greater decreases in SAD symptoms, and that increases in cognitive reappraisal self-efficacy during CBT mediated the effect of CBT on SAD symptom level. In children, general self-efficacy seem to mediate the association between negative self-statements and SAD symptoms (Rudy et al., 2011). In general, the literature suggests that changes in self-efficacy are associated with a decrease in SAD symptoms. However, as for PD, these studies did not investigate the role of treatment self-efficacy.

Research on the role of self-efficacy in Internet-delivered interventions is scarce. Most of this research focus on Internet-delivered interventions aimed at increasing self-efficacy, i.e., with self-efficacy as a treatment outcome, and not as a predictor of treatment outcome. The literature propose that self-efficacy can be effectively enhanced through Internet-delivered interventions in clinical populations and in non-clinical populations (Poddar et al., 2010; Ebert et al., 2014; Cieslak et al., 2016; Chao et al., 2019; Newby et al., 2020). To the best of our knowledge, no studies have investigated the role of treatment self-efficacy as a potential predictor of treatment outcomes in PD and SAD in Internet-delivered interventions.

In search of predictors of treatment outcomes, it is hypothesized that treatment self-efficacy might be a predictor of symptom reduction and dropout. It can be argued that guided Internet-delivered therapy, compared to traditional face-to-face CBT, requires more personal responsibility and motivation for commencing and completing treatment tasks as the therapeutic contact is reduced (Haug et al., 2015). This could imply that treatment self-efficacy potentially could be a predictor of symptom reduction and dropout in guided Internet-delivered therapy. A chief ingredient in the treatment of anxiety disorders is some form of exposure where the patient is encouraged to seek and expose themselves to that stimulus which they are most scared of. This naturally entails a fear response in the patients, which motivates and urges them to avoid this stimulus. Although exposure techniques are effective in the treatment of anxiety disorders, it can be argued that such treatment might be one of the most challenging treatments for patients as they demand that patients seek and sustain their most anxiety provoking fears. The challenging nature of exposure techniques could imply that treatment self-efficacy plays an especially central role in predicting symptom reduction and dropout in anxiety disorders.

The potential predictive value of assessing treatment self-efficacy at pre-treatment in guided Internet-delivered therapy for PD and SAD is, to the best of our knowledge, unexplored. The presented literature indicates that more knowledge regarding predictors of treatment outcome in guided Internet-delivered therapy is warranted. This study aims to examine if treatment self-efficacy is a predictor of symptom reduction and dropout in a relatively large sample (*N* = 575) of adult patients receiving guided ICBT for PD and SAD in a routine care setting. This study includes the following hypotheses:

1. High treatment self-efficacy predicts lower dropout rates.
2. High treatment self-efficacy predicts greater symptom reduction.

## Method

### Setting

This study was an open effectiveness study with a within-group design with repeated measures of outcome measures and 6-month follow-up conducted at three outpatient clinics in Bergen, Norway.

The clinic that delivers the guided Internet-delivered therapy that the participants in this study received is anonymized due to ethical concerns. This clinic offers guided ICBT-based treatment for PD, SAD, and depression in Norway. The clinic has offered ICBT for PD and SAD since 2013, while ICBT for depression has been offered since 2015 (Nordgreen et al., 2018b). The hospital which the clinic is affiliated with has a catchment area of 250,000 persons and comprises three mental health outpatient clinics. The Western Regional Committee for Medical and Health Research Ethics approved this study (reference number 2012/2211) and all participants signed informed consents before participating in the study.

All patients that receive specialized mental health treatment in Norway must be referred by their General Practitioner. Referred patients that were admitted for treatment were invited to a face-to-face assessment at their respective clinics. In this assessment, all patients were informed that guided ICBT was an available treatment format.

All patients that were referred to one of the three outpatient clinics for either SAD or PD and considered guided ICBT as a treatment format were invited to a diagnostic assessment with Mini-International Neuropsychiatric Interview (MINI). MINI is a widely used brief structured diagnostic interview for the major mental disorders in Diagnostic and Statistical Manual of Mental Disorders (DSM-IV) and International Classification of Diseases and related disorders (ICD-10) (World Health Organization, 1993; American Psychiatric Association, 1994; Sheehan et al., 1998). The administration time is ~15 min, and studies are reporting excellent interrater reliability and very good test-retest reliability (Sheehan et al., 1998). All patients who wished to start guided ICBT and met the inclusion criteria were offered guided ICBT and invited to participate in this study.

The inclusion criteria were: (1) PD or SAD had to be the main problem according to the results from MINI; (2) participants had to be 18 years or older; (3) no daily use of benzodiazepines; (4) if using antidepressants, the usage had to be stable for the past 4 weeks; (5) participants had to be able to read and write in Norwegian; (6) the guided Internet-delivered therapy delivered through this study should be the only treatment the participants receive during the research period. The exclusion criteria were: (1) current suicidal ideation; (2) current psychosis; (3) current substance abuse; (4) in immediate need of other treatment due to another acute and more severe condition; (5) no Internet access.

The treatment has the same elements as traditional face-to-face CBT, i.e., the same information, tools and techniques—the key difference is that the treatment and feedback is given *via* Internet (Helse Bergen, 2020). The treatment may last up to 14 weeks with the patients spending an average of 7–10 days per module. The participants gain access to each module upon finishing the previous one. Each treatment consists of nine chapters or modules with text and tasks. Each module takes ~45 min to complete, and the participant is given a week to complete each one. All participants have contact with their therapist electronically through messages at least once a week. The therapist's task is to provide guidance and support through the program. Therapist guidance is given at least once a week through a secure email system with an average of 10–15 min per week per patient (Nordgreen et al., 2018a). The therapists had educational backgrounds as nurses, social workers, clinical psychologists, and psychiatrists. There were 8 therapists in total, where 5 of them were females. The therapists' age span were 30–67 years. All therapists had participated in 1 year of continued education in guided ICBT. All therapists worked co-located 1 day each week to work with guided Internet-delivered therapy, providing a low threshold for seeking advice from each other. Challenges and successes from the therapy were discussed in this setting. An expert in guided ICBT provided weekly supervision. The therapists had an ordinary workload with face-to-face therapies the remaining days of the week. The first five modules are considered to be the most vital ones as this is where the participants are introduced to exposure therapy and obtain the necessary knowledge and skills in order to be able to execute exposure therapy themselves. The content covered in the first five modules for both PD and SAD consists of psychoeducation, working with automatic thoughts, and behavioral experiments (Nordgreen et al., 2018a). From a clinical viewpoint and based on shared experiences from the therapists delivering the guided Internet-delivered therapy, many patients show rapid improvement after the employment of exposure techniques. The last four modules consist of repetition of what is previously learned, more exposure and relapse prevention.

The treatment for PD builds on research from Sweden and is an adapted version of a Swedish ICBT-program, which was translated to Norwegian in 2007 and revised in 2012 (Carlbring et al., 2006). Previous studies on the guided ICBT version used in the current study have reported large effect sizes for PD (Nordgreen et al., 2015, 2018b). The ICBT for PD is comprised of nine modules that all participants gained access to *via* the Internet. The treatment is based on the work of Clark (1986) and the modules include elements as psychoeducation, CBT-procedures for identifying and changing automatic thoughts, behavioral experiments, *in-vivo* exposure and relapse prevention (Carlbring et al., 2006).

The treatment for SAD builds on research from Sweden (Furmark et al., 2009; Hedman et al., 2014). Previous studies on the guided ICBT version used in the current study have reported large effect sizes for SAD (Nordgreen et al., 2015; Nordmo et al., 2015). The ICBT for SAD consists of nine modules that all participants gained access to *via* the Internet. The program include elements such as psychoeducation, changing automatic thoughts, behavioral experiments, shifting focus, and relapse prevention (Nordgreen et al., 2018a).

### Participants

A total of 605 patients who received treatment for PD or SAD agreed to participate in this study. Thirty patients were excluded from the analyses because they had more than one unanswered item on the treatment self-efficacy scale, resulting in 575 patients being included in the analyses, where 280 pertained to the PD treatment and 306 to the SAD treatment. The descriptive data of the participants are reported in Table 1. The mean age was 31.8 (*SD* = 10.9), and there were 61.4% females (*N* = 351). In the PD treatment, the mean age was 34.2 (*SD* = 11.2) and there were 65.3% females (*N* = 179). In the SAD treatment, the mean age was 29.6 (*SD* = 10), and there were 57.7% females (*N* = 172). 42.5% (*N* = 242) in the total sample were single, 54.4% (*N* = 310) were married or partnered, and 3% (*N* = 17) were divorced or widowed. In the PD treatment 30.8% were single (*N* = 84), 65.6% were married or partnered (*N* = 179), and 3.6% (*N* = 10) were divorced or widowed. In The SAD treatment 53.4% (*N* = 158) were single, 44.3% (*N* = 131) were married or partnered, and 2.4% (*N* = 7) were divorced or widowed. In the total sample 37.1% *(N* = 212) had children. In the PD treatment 48.2% (*N* = 132) had children, while 26.8% (*N* = 80) had children in the SAD treatment. In the total sample 39.9% (*N* = 228) had higher education, i.e., college or university level. In the PD treatment 42.7% (*N* = 117) had higher education, while 37.2% (*N* = 111) had higher education in the SAD treatment. All patients who received Guided ICBT for PD or SAD at outpatient clinics at Haukeland University Hospital between 2014 and 2019 were invited to participate in this study.

**Table 1:** Demographic data.

	**Total sample**		**PD treatment**		**SAD treatment**	
	***N***	**%**	***N***	**%**	***N***	**%**
**Gender**						
Female	351	61.4	179	65.3	172	57.7
Male	221	38.6	95	34.7	126	42.3
**Marital status**						
Single	242	42.5	84	30.8	158	53.4
Married/partnered	310	54.4	179	65.6	131	44.3
Divorced/widowed	17	3	10	3.6	7	2.4
Children^a^	212	37.1	132	48.2	80	26.8
Higher education^b^	228	39.9	117	42.7	111	37.2

*N, number of participants. N = 575 (n = 280 in the PD treatment and n = 306 in the SAD treatment). Eleven participants received treatment for both PD and SAD, which explains why n in the PD treatment and n in SAD treatment exceeds the N in the total sample*.

a *Reflects the number and percentage of participants answering “yes” to this question*.

b *College or university level*.

### Measures

All measures (sociodemographic questionnaire, anxiety symptom measures, and treatment self-efficacy) applied in this study were integrated in the platform where the participants received treatment. All measures were applied pre-treatment, while only the anxiety symptom measures were applied throughout the treatment, post-treatment and at follow-up.

#### Predictor

##### Bergen Genetic Counseling Self-Efficacy Scale

The treatment self-efficacy measure is based on the Bergen Genetic Counseling Self-Efficacy Scale, which was developed using Bandura's Guide for Constructing Self-efficacy Scales (Bandura, 2006; Bjorvatn et al., 2009). Treatment self-efficacy was measured at baseline before starting treatment. The Bergen Genetic Counseling Self-Efficacy Scale has excellent internal consistency (Cronbach's alpha = 0.95) and was adapted to a guided Internet-delivered therapy format. The adapted scale also has an excellent internal consistency (Cronbach's alpha = 0.91) and constitutes 15 items which are scored from one (*can certainly not cope*) to 10 (*can certainly cope*). The questionnaire is attached in the appendix.

#### Outcomes

##### Body Sensations Questionnaire

BSQ is a 17-item self-report questionnaire with items concerning sensations associated with autonomic arousal (Chambless et al., 1984). Each item is rated on a five-point scale, ranging from one *(not frightened or worried by this sensation)* to five *(extremely frightened by this sensation)*, indicating how anxiety provoking each sensation is to the patient. The total score is calculated by averaging all the item ratings. A high score reflects high fear of bodily symptoms. Previous research has found high internal consistency with a Cronbach's alpha of = 0.87, as well as a moderately good test-retest reliability of *r* = 0.67 (Chambless et al., 1984). BSQ was administered on 10 different occasions: at pre-treatment, after each of the modules two-nine, and at 6-month follow-up.

Social Phobia Scale (SPS). SPS is used to assess self-reported symptoms of SAD in performance situations, such as fears of being scrutinized during routine activities like eating, drinking, or writing (Mattick and Clarke, 1998). SPS has high levels of internal consistency for SAD (Cronbach's alpha = 0.89) and test-retest reliability (0.91 over 4 weeks and 0.93 over 12 weeks) (Mattick and Clarke, 1998). SPS correlates well with established measures of social anxiety. SPS is comprised of 20 items where each item is rated on a five-point scale, ranging from zero *(not at all characteristic or true for me)* to four *(extremely characteristic or true for me)*. The total score is calculated by summarizing all item scores, meaning that all total scores on SPS vary between zero and 80. A high score indicates a high level of symptoms. SPS was administered on 10 different occasions: at pre-treatment, after each of the modules two-nine, and at 6-month follow-up.

#### Sociodemographic Questionnaire

A sociodemographic questionnaire developed for this study was applied. The questionnaire collected typical background data such as gender, age, highest achieved educational level, profession, marital status, and number of children.

### Statistical Analyses

#### Self-Efficacy Principal Component Analysis

All statistical analyses were carried out in SPSS 26.0. We wished to investigate if certain elements of treatment self-efficacy had greater predictive properties than the entire construct of treatment self-efficacy. Therefore, it was decided to explore whether treatment self-efficacy had an underlying factorial structure and could be divided into meaningful subscales. We did this through the application of a principal component analysis with direct oblimin rotation on the 15 items in the Bergen Genetic Counseling Self-Efficacy Scale questionnaire. The results from this analysis, i.e., the subscales of treatment self-efficacy, was applied (in addition to the total score of treatment self-efficacy) in all subsequent analyses when the association between treatment self-efficacy and outcomes was examined. A Cronbach's alpha reliability test was run for each of the subscales to investigate their internal consistency.

#### Dropout Measures

In this study two dropout measures were included. The first, labeled “Number of modules completed,” describes how many participants completed each module. The second, labeled “Completed main parts of the program”, describes how many participants completed the main parts of the program (≥ five modules completed).

#### Treatment Self-Efficacy's Correlation With Dropout

To investigate if treatment self-efficacy was negatively correlated with dropout a Pearson product-moment correlation was utilized, correlating treatment self-efficacy (subscales included) with both dropout measures in the total sample, the PD treatment, and the SAD treatment.

#### Mixed Linear Model

To investigate if treatment self-efficacy predicted greater symptom reduction a mixed linear model (MLM) fitted with Full Information Maximum Likelihood (FIML), was applied. MLM is appropriate and recommended for longitudinal data with repeated measures (Hesser, 2015). MLMs incorporate both fixed effects (i.e., averages across individuals) and random effects (i.e., individual deviations from these averages), thus offering a flexible and appropriate method for analyzing complex data (Hesser, 2015). MLMs have multiple advantages over traditional approaches of analysis of repeated-measures data, such as the ability to incorporate time-varying predictors, handle dependence among repeated observations flexibly and to provide accurate estimates of missing data (Hesser, 2015). MLMs handle missing data by providing estimated means based on the available data. The application of FIML, which assumes that data are missing at random, was applied to handle the missing data, thus making it possible to include participants with missing data in the analyses (Little and Rubin, 2019). A multilevel, mixed model framework was applied in this study. A MLM was used to analyse the effect of treatment self-efficacy on the outcome measures (BSQ and SPS) across 10 measurement points.

Model selection was based on the best fit as indicated by likelihood ratio tests for nested models. The model with the best fit included fixed time and self-efficacy scores. Fixed predictors, or time-invariant covariates, account for the variance in outcome between individuals (Hesser, 2015). This is what we aimed to investigate, i.e., if the fixed predictor treatment self-efficacy account for the variance between individuals in symptom reduction. The main effects of time and treatment self-efficacy was examined. Interaction effects were analyzed with a fixed effect interaction between time and treatment self-efficacy.

In the MLM, the treatment self-efficacy scores were centered around their own average. The centered value is computed by subtracting the average of treatment self-efficacy from the observed value (centered value = observed value—average self-efficacy). This procedure makes it more apparent how individuals with deviant self-efficacy scores vary on the outcome measures, thus making it easier to examine if and how individuals with either high or low self-efficacy scores differ in symptom variance throughout the treatment.

## Results

### Descriptive Data

#### Self-Efficacy

The Kaiser-Meyer-Olkin Measure (KMO) of sampling adequacy yielded KMO = 0.88, i.e., meritorious, indicating that the sampling is adequate (Revelle, 2016). An analysis of eigenvalues for each factor was performed, showing that four factors had eigenvalues over Kaiser's criterion of 1, and in combination explained 76.7% of the total variance. The interpretation of the four components yielded the following pattern: (1) self-efficacy for prioritizing the treatment; (2) self-efficacy for seeking support during treatment (3) self-efficacy for using technical aids (4) self-efficacy for overcoming adversities during the treatment. The reliability test of the four subscales yielded the following Cronbach's alpha scores: 0.92 for factor one; 0.88 for factor two; 0.65 for factor three; and 0.89 for factor four. Factor one, two and four has good to excellent internal consistency, while factor three has a low internal consistency and thus should be interpreted with care. However, factor three has the lowest number of items (three items), which may lower the factor's internal consistency.

The descriptive data of treatment self-efficacy and the four subscales for the total sample, the PD treatment, and the SAD treatment are reported in Table 2. In the total sample the results yielded the following scores: total treatment self-efficacy (*M* = 7.76, *SD* = 1.34), self-efficacy for prioritizing the treatment (*M* = 7.75, *SD* = 1.67), self-efficacy for seeking support during treatment (*M* = 7.11, *SD* = 2.15), self-efficacy for using technical aids (*M* = 9.1, *SD* = 1.14), self-efficacy for overcoming adversities during the treatment (*M* = 7.44, *SD* = 1.6). In the PD treatment the results yielded the following scores: total treatment self-efficacy (*M* = 8.15, *SD* = 1.23), self-efficacy for prioritizing the treatment (*M* = 8.06, *SD* = 1.57), self-efficacy for seeking support during treatment (*M* = 7.79, *SD* = 1.88), self-efficacy for using technical aids (*M* = 9.22, *SD* = 1.13), self-efficacy for overcoming adversities during the treatment (*M* = 7.78, *SD* = 1.54). In the SAD treatment the results yielded the following scores: total treatment self-efficacy (*M* = 7.39, *SD* = 1.33), self-efficacy for prioritizing the treatment (*M* = 7.46, *SD* = 1.71), self-efficacy for seeking support during treatment (*M* = 6.47, *SD* = 2.19), self-efficacy for using technical aids (*M* = 8.92, *SD* = 1.13), self-efficacy for overcoming adversities during the treatment (*M* = 7.11, *SD* = 1.59).

**Table 2:** Descriptive data of the self-efficacy scale.

	***N***	***M***	***SD***	**Min**	**Max**
**Total sample**					
Total Sef	573	7.76	1.34	1	10
Sef prioritizing	574	7.75	1.67	1	10
Sef support	575	7.11	2.15	1	10
Sef technical aids	574	9.1	1.14	1	10
Sef adversities	575	7.44	1.6	1	10
**PD treatment**					
Total Sef	276	8.15	1.23	1	10
Sef prioritizing	276	8.06	1.57	1	10
Sef support	277	7.79	1.88	1	10
Sef technical aids	277	9.22	1.13	1	10
Sef adversities	277	7.78	1.54	1	10
**SAD treatment**					
Total Sef	297	7.39	1.33	3.4	10
Sef prioritizing	298	7.46	1.71	2	10
Sef support	298	6.47	2.19	1.5	10
Sef technical aids	297	8.92	1.13	4.67	10
Sef adversities	298	7.11	1.59	2.75	10

*N, number of participants; M, mean; SD, standard deviation; Min, minimum value; Max, maximum value; Sef, treatment self-efficacy*.

An independent samples *t*-test was conducted to examine potential differences in levels of treatment self-efficacy between the PD and SAD treatments. There were statistically significant differences in total self-efficacy between the PD treatment (*M* = 8.15, *SD* = 1.23) and the SAD treatment [*M* = 7.39, *SD* = 1.33; *t*(571) = 7.07, *p* < 0.001]. The magnitude of the differences in the means (mean difference = 0.76, 95% CI [0.55 −0.97]) was small (eta squared = 0.08).

#### Anxiety Symptom Levels

The descriptive data of anxiety symptom levels throughout the treatment for PD and SAD are reported in Table 3. In the PD treatment the following scores were reported: pre-treatment (*N* = 241, *M* = 2.66, *SD* = 0.7); module 2 (*N* = 222, *M* = 2.59, *SD* = 0.76); module 3 (*N* = 203, *M* = 2.42, *SD* = 0.78); module 4 (*N* = 183, *M* = 2.23, *SD* = 0.75); module 5 (*N* = 161, *M* = 2.05, *SD* = 0.7); module 6 (*N* = 151, *M* = 1.98, *SD* = 0.73); module 7 (*N* = 129, *M* = 1.87, *SD* = 0.69); module 8 (*N* = 126, *M* = 1.8, *SD* = 0.71); module 9 (*N* = 104, *M* = 1.7, *SD* = 0.67); and 6-month follow-up (*N* = 91, *M* = 1.62, *SD* = 0.61).

**Table 3:** Descriptive data of anxiety symptom levels throughout the treatment.

	***N***	***M***	***SD***	**Min**	**Max**
**PD treatment**					
Module 0	241	2.66	0.7	1.06	4.31
Module 1					
Module 2	222	2.59	0.76	1	4.38
Module 3	203	2.42	0.78	1.06	4.75
Module 4	183	2.23	0.75	1	4.25
Module 5	161	2.05	0.7	1	4.13
Module 6	151	1.98	0.73	1	3.81
Module 7	129	1.87	0.69	1	3.63
Module 8	126	1.8	0.71	1	4.44
Module 9	104	1.7	0.67	1	3.5
FU-6	91	1.62	0.61	1	3.5
**SAD treatment**					
Module 0	295	39.15	15.45	7	80
Module 1					
Module 2	249	39.21	15.8	5	77
Module 3	218	35.61	15.39	6	78
Module 4	192	33.09	16.11	3	79
Module 5	166	31.3	15.87	1	71
Module 6	142	28.44	15.8	0	71
Module 7	127	27.62	15.92	1	74
Module 8	117	25.75	16.42	0	71
Module 9	104	23.13	15.97	0	66
FU-6	82	23.39	15.63	1	59

*N, number of participants; M, mean; SD, standard deviation; Min, minimum value; Max, maximum value; FU-6, 6-month follow-up*.

In the SAD treatment the following scores were reported: pre-treatment (*N* = 295, *M* = 39.15, *SD* = 15.45); module 2 (*N* = 249, *M* = 39.21, *SD* = 15.8); module 3 (*N* = 218, *M* = 35.61, *SD* = 15.39); module 4 (*N* = 192, *M* = 33.09, *SD* = 16.11); module 5 (*N* = 166, *M* = 31.3, *SD* = 15.87); module 6 (*N* = 142, *M* = 28.44, *SD* = 15.8); module 7 (*N* = 127, *M* = 27.62, *SD* = 15.92); module 8 (*N* = 117, *M* = 25.75, *SD* = 16.42); module 9 (*N* = 104, *M* = 23.13, *SD* = 15.97); and 6-month follow-up (*N* = 82, *M* = 23.39, *SD* = 15.63).

#### Dropout

The descriptive data of the two dropout measures are presented in Table 4. In the total sample 57% (*N* = 327) completed the main parts of the program. In the PD treatment 58.1% (*N* = 161) completed the main parts of the program, while 55.7% (*N* = 166) in the SAD treatment completed the main parts of the program. In the total sample 93.2% (*N* = 536) completed the first module, and 30.4% (*N* = 175) completed the 6-month follow-up. The remaining modules showed the following pattern: module 2: 81.9% (*N* = 471); module 3: 73.4% (*N* = 422); module 4: 65.4% (*N* = 376); module 5: 57% (*N* = 328); module 6: 51.1% (*N* = 294); module 7: 45% (*N* = 259); module 8: 42.6% (*N* = 245), module 9: 36.5% (*N* = 210).

**Table 4:** Descriptive data of dropout.

	**Total sample**		**PD treatment**		**SAD treatment**	
	***N***	**%**	***n***	**%**	***n***	**%**
**Completed main parts**						
Yes	327	57	161	58.1	166	55.7
No	248	43	116	41.9	132	44.3
**No. modules completed**						
Module 0	536	93.2	244	87	303	99
Module 1						
Module 2	471	81.9	225	80.4	257	84
Module 3	422	73.4	206	73.6	226	73.9
Module 4	376	65.4	186	66.4	200	65.4
Module 5	328	57	164	58.6	173	56.5
Module 6	294	51.1	154	55	149	48.7
Module 7	259	45	132	47.1	135	44.1
Module 8	245	42.6	128	45.7	122	39.9
Module 9	210	36.5	106	37.9	108	35.3
FU-6	175	30.4	92	32.9	85	27.8

*N = 575 (n = 280 in the PD treatment and n = 306 in the SAD treatment), FU-6 = 6-month follow-up*.

In the PD treatment 87% (*N* = 244) completed the first module, and 32.9% (*N* = 92) completed the six-month follow-up. The remaining modules showed the following pattern: module 2: 80.4% (*N* = 225); module 3: 73.6% (*N* = 206); module 4: 66.4% (*N* = 186); module 5: 58.6% (*N* = 164); module 6: 55% (*N* = 154); module 7: 47.1% (*N* = 132); module 8: 45.7% (*N* = 128); module 9: 37.9% (*N* = 106).

In the SAD treatment 99% (*N* = 303) completed the first module, and 27.8% (*N* = 85) completed the 6-month follow-up. The remaining modules showed the following pattern: module 2: 84% (*N* = 257); module 3: 73.9% (*N* = 226); module 4: 65.4% (*N* = 200); module 5: 56.5% (*N* = 173); module 6: 48.7% (*N* = 149); module 7: 44.1% (*N* = 135); module 8: 39.9% (*N* = 122), module 9: 35.3% (*N* = 108).

### The Association Between Treatment Self-Efficacy and Dropout

The correlation between treatment self-efficacy and the two dropout measures for the total sample, the PD treatment, and the SAD treatment are shown in Table 5.

**Table 5:** Pearson correlation matrix between self-efficacy and dropout.

**Variable**	**Total sef**	**Sef for prioritizing**	**Sef for seeking support**	**Sef for technichal aids**	**Sef for overcoming adversities**
**Total sample**					
Completed main parts	0.13^**^	0.13^**^	0.07	0.1^*^	0.11^**^
No. modules completed	0.16^**^	0.16^**^	0.1^*^	0.11^**^	0.14^**^
**PD treatment**					
Completed main parts	0.07	0.13^*^	0.01	0.05	0.04
No. modules completed	0.09	0.15^*^	0.04	0.06	0.03
**SAD treatment**					
Completed main parts	0.17^**^	0.12^*^	0.11	0.14^*^	0.17^**^
No. modules completed	0.22^**^	0.16^**^	0.14^*^	0.18^**^	0.22^**^

* *p < 0.05*,

** *p < 0.01, Sef, treatment self-efficacy*.

In the total sample there were significant positive correlations between all self-efficacy measures and the number of modules completed: total self-efficacy, *r* = 0.16, *n* = 573, *p* = < 0.001; self-efficacy for prioritizing the treatment, *r* = 0.16, *n* = 574, *p* = < 0.001; self-efficacy for seeking support during treatment, *r* = 0.1, *n* = 575, *p* = 0.02; self-efficacy for using technical aids, *r* = 0.11, *n* = 574, *p* = 0.009; and self-efficacy for overcoming adversities during the treatment, *r* = 0.14, *n* = 575, *p* = < 0.001. Significant positive correlations between all the self-efficacy measures and completed main parts were also found, except for the correlation between the subscale self-efficacy for seeking support and completed main parts: total self-efficacy, *r* = 0.13, *n* = 573, *p* = 0.002; self-efficacy for prioritizing the treatment, *r* = 0.13, *n* = 574, *p* = 0.002; self-efficacy for seeking support during treatment, *r* = 0.07, *n* = 575, *p* = 0.096; self-efficacy for using technical aids, *r* = 0.1, *n* = 574, *p* = 0.016; and self-efficacy for overcoming adversities during the treatment, *r* = 0.11, *n* = 575, *p* = 0.007.

In the PD treatment there was a significant positive correlation between self-efficacy for prioritizing the treatment and number of completed modules, *r* = 0.15, *n* = 276, *p* = 0.016. A significant positive correlation between self-efficacy for prioritizing the treatment and completed main parts was also found, *r* = 0.13, *n* = 276, *p* = 0.029. The rest of the results were not significant.

In the SAD treatment there were significant positive correlations between all self-efficacy scores and both dropout measures—the only exception was the correlation between self-efficacy for seeking support and completed main parts of the program, which was not significant. The correlations between number of completed modules and the respective self-efficacy scores were: total self-efficacy, *r* = 0.22, *n* = 297, *p* = < 0.001; self-efficacy for prioritizing the treatment, *r* = 0.16, *n* = 298, *p* = 0.005; self-efficacy for seeking support during treatment, *r* = 0.14, *n* = 298, *p* = 0.018; self-efficacy for using technical aids, *r* =0.18, *n* = 297, *p* =0.002; and self-efficacy for overcoming adversities during the treatment, *r* = 0.22, *n* = 298, *p* = < 0.001. The correlations between completed main parts and the respective self-efficacy scores were: total self-efficacy, *r* = 0.17, *n* = 297, *p* = 0.003; self-efficacy for prioritizing the treatment, *r* = 0.12, *n* = 298, *p* = 0.034; self-efficacy for seeking support during treatment, *r* = 0.11, *n* = 298, *p* = 0.064; self-efficacy for using technical aids, *r* = 0.14, *n* = 297, *p* = 0.02; and self-efficacy for overcoming adversities during the treatment, *r* = 0.17, *n* = 298, *p* = 0.003.

It should be noted that all the significant results were small in effect size, i.e., *r* = < 0.3, indicating that the association between treatment self-efficacy and dropout is weak, although it is significant (Cohen, 1992).

### The Association Between Treatment Self-Efficacy and Symptom Reduction

#### Self-Efficacy and Symptom Reduction in the PD Treatment

The results from the MLM analysis for the PD treatment are presented in Table 6. The MLM analysis showed significant main effects of treatment self-efficacy on symptom reduction (*p* = 0.017), and of time on symptom reduction (*p* < 0.001). The overall interaction effect of time and treatment self-efficacy was not significant (*p* = 0.155). However, significant interaction effects were found for some of the individual modules in the treatment program.

**Table 6:** Results of MLM analysis of treatment self-efficacy and symptomatic reduction.

	***β***	**SE**	***p***	***CI*** **(95%)**	***β*** **time x Sef**	***p*** **time x Sef**
**PD treatment**						
Intercept	2.69	0.47	0.00^**^	2.59 to 2.78	−0.07	0.07
Module 1	−0.06	0.36	0.11	−0.13 to 0.01	−0.09	0.01^**^
Module 3	−0.25	0.38	0.00^**^	−0.32 to −0.17	−0.14	0.00^**^
Module 4	−0.42	0.39	0.00^**^	−0.5 to −0.35	−0.09	0.03^*^
Module 5	−0.63	0.41	0.00^**^	−0.71 to −0.55	−0.07	0.07
Module 6	−0.72	0.43	0.00^**^	−0.8 to−0.63	−0.07	0.09
Module 7	−0.8	0.45	0.00^**^	−0.89 to−0.71	−0.07	0.08
Module 8	−0.85	0.46	0.00^**^	−0.94 to −0.76	−0.07	0.1
Module 9	−0.97	0.48	0.00^**^	−1.06 to −0.87	−0.04	0.32
6M-FU	−0.99	0.51	0.00^**^	−1.09 to −0.88	−0.05	0.13
**SAD treatment**						
Intercept	38.82	0.95	0.00^**^	36.95 to 40.7	−1.5	0.04^*^
Module 1	0.13	0.64	0.846	−1.14 to 1.39	−1.14	0.12
Module 3	−2.64	0.67	0.00^**^	−3.95 to −1.32	−1.31	0.08
Module 4	−5.01	0.7	0.00^**^	−6.38 to −3.65	−2.15	0.01^**^
Module 5	−6.68	0.73	0.00^**^	−8.12 to 5.25	−2.23	0.01^**^
Module 6	−8.72	0.77	0.00^**^	−10.22 to −7.21	−2.8	0.00^**^
Module 7	−9.15	0.79	0.00^**^	−10.71 to −7.59	−2.22	0.01^**^
Module 8	−11.41	0.82	0.00^**^	−13.02 to −9.8	−2.2	0.01^**^
Module 9	−13.88	0.86	0.00^**^	−15.56 to −12.2	−1.36	0.13
6M-FU	−14.2	0.94	0.00^**^	−16.04 to −12.36	−1.05	0.13

*β, parameter estimates; SE, standard error; CI, confidence interval; Sef, treatment self-efficacy; β Time x Sef, parameter estimates of the interaction effect between time and treatment self-efficacy; p time x Sef, p-value of the interaction effect between time and treatment self-efficacy;*

* *p < 0.05*.

** *p < 0.01*.

The significant main effect of treatment self-efficacy on symptom reduction occurred already after module three and persisted for all the later modules. The results indicated that a patient with an average level of total self-efficacy after module three showed a symptom reduction of −0.25 (*p* < 0.001, 95% CI [−0.32, −0.17]) on the BSQ score, and the reduction was consistently increasing for all later modules.

Significant interaction effects of treatment self-efficacy and time on symptom reduction was found after module one (β = −0.09, *p* < 0.001, 95% CI [−0.14 to 0.01]), three (β = −0.14, *p* < 0.001, 95% CI [−0.22, −0.07]) and four (β = −0.09, *p* = 0.03, 95% CI [−0.16, −0.01]), meaning that those with a higher total self-efficacy score showed greater symptom reduction after these modules beyond the main effect of self-efficacy on symptom reduction.

In the PD treatment, MLM was also run for each of the subscales of self-efficacy to investigate their separate effect on symptom reduction. Each subscale had, like treatment self-efficacy, significant main effects of both time and the subscales of self-efficacy on symptom reduction after every module with increasing effect per module. The only exception was after module 1 where no significant main effects were found. Interaction effects of time and each subscale of self-efficacy on symptom reduction were also investigated. All the significant interaction effects were related to after module three: self-efficacy for prioritizing the treatment and symptom reduction (β = −0.077, *p* < 0.001, 95% CI [−0.12, −0.03]), self-efficacy for seeking support during treatment and symptom reduction (β = −0.062, *p* = 0.002, 95% CI [−0.1, −0.02]), and self-efficacy for overcoming adversities during the treatment and symptom reduction (β = −0.05, *p* = 0.037, 95% CI [−0.1, 0.0]).

#### Self-Efficacy and Symptom Reduction in the SAD Treatment

The results from the MLM analysis for SAD are presented in Table 6. The MLM analysis showed significant main effects of both treatment self-efficacy on symptom reduction (*p* = 0.007), and for time on symptom reduction (*p* < 0.001). The overall interaction effect of time and treatment self-efficacy was not significant (*p* = 0.11). However, significant interaction effects were found after several of the individual modules in the treatment program.

The significant main effect of treatment self-efficacy on symptom reduction occurred already after module three and persisted for all the later modules. The results indicated that a patient with an average level of total self-efficacy after module three showed a symptom reduction of −2.6 (*p* < 0.001, 95% CI [−3.95, −1.32]) on the SPS score, and the reduction was consistently increasing for all later modules.

Significant interaction effects of treatment self-efficacy and time on symptom reduction was found at intercept (β = −1.5, *p* = 0.039, 95% CI [−2.92, −0.75]) and after module four (β = 2.15, *p* = 0.006, 95% CI [−3.67, −0.62]), five (β = −2.23, *p* = 0.006, 95% CI [−3.8, −0.65]), six (β = −2.8, *p* = 0.001, 95% CI [−4.4, −1.18]), seven (β = −2.22, *p* = 0.008, 95% CI [−3.86, −0.57]), and eight (β = −2.2, *p* = 0.01, 95% CI [−3.88, −0.53]), meaning that those with a higher total self-efficacy score showed a greater symptom reduction after these modules beyond the main effect of self-efficacy on symptom reduction.

MLM was also run for each of the subscales of self-efficacy. Each subscale had, like treatment self-efficacy, significant main effects of both time and the subscales of treatment self-efficacy on symptom reduction after every module with increasing effect per module. The only exception was after module one where no significant main effects were found. Interaction effects of each subscale of self-efficacy and time on symptom reduction were also investigated. The significant interaction effects were between self-efficacy for seeking support during treatment and symptom reduction after module five (β = −0.97, *p* = 0.004, 95% CI [−1.63, −0.31]), six (β = −0.88, *p* = 0.012, 95% CI [−1.56, −0.19]), seven (β = −1.16, *p* < 0.001, 95% CI [−1.86, −0.46]), eight (β = −0.82, *p* = 0.022, 95% CI [−1.55, −0.12]) and nine (β = −0.89, *p* = 0.019, 95% CI [−1.64, −0.15]); between self-efficacy for using technical aids and symptom reduction at intercept (β = −1.68, *p* = 0.039, 95% CI [−3.27, −0.08]) and after module seven (β = −1.9, *p* = 0.023, 95% CI [−3.53, −0.27]); and between self-efficacy for overcoming adversities during the treatment and symptom reduction at intercept (β = −1.32, *p* = 0.024, 95% CI [−2.47, −0.17]).

## Discussion

This study examined the role of treatment self-efficacy in an open effectiveness study of guided ICBT for PD and SAD in Norway. The focus of the study was to examine whether high treatment self-efficacy was a predictor of lower dropout rates and greater symptom reduction in guided Internet-delivered therapy.

The results support the first hypothesis that high treatment self-efficacy predicts lower dropout rates in PD and SAD. However, all the significant effect sizes were small. Only one subscale correlated significantly with the two dropout measures in PD (self-efficacy for prioritizing the treatment), while all the correlations but one was significant in SAD; the correlation between self-efficacy for seeking support and completed main parts was not significant. The results indicate that high treatment self-efficacy might be a predictor of lower dropout rates in both PD and SAD—but a stronger predictor of dropout in SAD. It is unclear why the association between treatment self-efficacy and dropout varied as much as it did between the two treatments. The PD and SAD treatments differed in several of the demographic variables: the PD treatment included more females, more patients who were married or had a partner, more patients with children, more patients with higher education, and the mean age was higher, compared to the SAD treatment. Beatty and Binnion (2016) found in their review that being female predicted higher adherence in unguided Internet-delivered interventions for a wide range of mental disorders, which is contrary to the results from this study when you compare the PD and SAD treatment. El Alaoui et al. (2015b) on the other hand found that male gender predicted lower adherence in ICBT for SAD. Hadjistavropoulos et al. (2015) found that high age and high education predicted low levels of dropout in guided ICBT for depression and generalized anxiety disorder. The sample in the SAD treatment was younger and had slightly less highly educated patients compared to the PD treatment, which is contrary to what Hadjistavropoulos et al. (2015) found. However, the mentioned studies did not investigate treatment self-efficacy as a predictor, thus making a direct comparison of results difficult. The research on predictors of dropout and adherence is characterized by inconsistent results, which complicates the interpretation of the differences in predictors of dropout between the PD and SAD treatment.

The results support the second hypothesis, that high treatment self-efficacy predicts greater symptom reduction. The results showed significant main effects of both treatment self-efficacy and time (i.e., staying active in the treatment over the measurement points) on symptom reduction, with significant results after every module for both the PD and the SAD treatment, apart from after module one. This is in line with previous research on self-efficacy and anxiety disorders in face-to-face CBT, which suggest that high self-efficacy is associated with a decrease in PD and SAD symptoms (Telch et al., 1989; Richards et al., 2002; Casey et al., 2005; Goldin et al., 2012; Iancu et al., 2015). For example, Iancu et al. (2015) found that high general self-efficacy predicted lower symptom severity in SAD patients compared to a healthy control group. However, there is also evidence indicating that lower levels of self-efficacy predict greater symptom reduction. A study that classified insomnia patients receiving CBT into two groups based on their sleep-related beliefs (sleep-related self-efficacy among them) found that the group with lower sleep-related self-efficacy responded best to treatment (Edinger et al., 2008).

The results also indicate that patients with high treatment self-efficacy might benefit more from guided Internet-delivered therapy. After some specific modules, significant interaction effects of time and treatment self-efficacy were found, although the overall interaction effect was not significant in the PD treatment (*p* = 0.155) nor in the SAD treatment (*p* = 0.11). The results suggest that treatment self-efficacy, at certain parts of the treatment, acted as a moderator of symptom reduction in guided Internet-delivered therapy for PD and SAD. It is hard to interpret this result as existing research on potential moderators of symptom reduction in PD and SAD is limited, particularly research on self-efficacy as a moderator and guided Internet-delivered therapy as the treatment format. Lower levels of anxiety and depression symptoms has been found to moderate symptom reduction, yielding higher symptom reduction in ICBT but not in CBT for SAD patients (Hedman et al., 2012). More research exists on self-efficacy as a mediator or non-specific predictor in anxiety disorders, indicating that various types of self-efficacy might mediate symptom reduction in PD and SAD (Casey et al., 2004; Gallagher et al., 2013; Fentz et al., 2014; Iancu et al., 2015). Due to the lack of research on self-efficacy as a moderator of treatment outcome in guided Internet-delivered therapy, this result should be interpreted with care.

As already mentioned, increased specificity in operationalizations of self-efficacy is associated with increased predictive properties. With this in mind, we also examined the association between the outcome measures and the four subscales of treatment self-efficacy. Overall, the subscales of self-efficacy neither predicted dropout nor symptom reduction better than the total score of treatment self-efficacy. None of the significant correlations between the subscales and dropout were stronger than the correlation between the total score of treatment self-efficacy and dropout. None of the significant associations from the MLM analysis between the subscales and symptom reduction were stronger than the associations between the total score of treatment self-efficacy and symptom reduction. However, the results showed that self-efficacy for seeking support during treatment interacted frequently throughout the treatment with time on symptom reduction in SAD, as significant interaction effects were found after module five, six, seven, eight, and at post-treatment. The interaction effects of the total score of treatment self-efficacy and symptom reduction occurred at intercept after module four, five, six, seven and eight, i.e., at one more measurement point than self-efficacy for seeking support during treatment. This finding suggests that although self-efficacy for seeking support during treatment did not predict nor moderate symptom reduction as well as the total score of treatment self-efficacy, the subscale seems to cover the most essential parts of treatment self-efficacy in predicting symptom reduction in SAD.

An important notion to bear in mind when comparing the results between the PD and SAD treatment is the differences in levels of treatment self-efficacy between the two treatments. The PD treatment had a significantly higher mean of total self-efficacy than the SAD treatment (although the difference was small), something which could have an impact on the results. It could be argued that this is somewhat surprising as treatment self-efficacy seemed to be a predictor of successful treatment outcomes regardless of treatment, but a stronger predictor in SAD than PD. This is the case despite the PD treatment having a higher mean of treatment self-efficacy. One could then maybe expect that the differences between the treatments would have been larger with identical means of treatment self-efficacy across treatments. This, however, is only a hypothesis which needs to be examined further do draw any conclusions.

### Limitations

There are several limitations in the current study that need to be addressed. First, no comparison condition was included in the study, which prevents definitive conclusions about whether treatment self-efficacy is a specific mechanism of guided Internet-delivered therapy or not. This study did not compare two treatment forms, which makes the comparison of treatment self-efficacy as a moderator in different treatments impossible. When the knowledge of moderators of treatment outcome is applied in a routine care setting it is typically done by assisting clinicians in allocating patients to one of the multiple available treatments they are most likely to benefit from. However, this study did not compare the role of treatment self-efficacy in ICBT with the role of treatment self-efficacy in another treatment, e.g., CBT. This makes it difficult to determine whether high treatment self-efficacy makes a patient more appropriate for guided ICBT than face-to-face CBT, based on the results from this study. What we do know, based on the results from this study, is that patients with high treatment self-efficacy are likely to benefit from guided ICBT. Second, although the results suggest that treatment self-efficacy could be a moderator of treatment outcome in guided Internet-delivered therapy, this was only the case at specific parts of the treatment—the overall interaction effect of time and treatment self-efficacy was not significant in both the PD treatment (*p* = 0.155) and the SAD treatment (*p* = 0.11). Third, the dropout rate was relatively high, which might have had an impact on the validity of the results. High dropout rates result in much missing data in the later parts of the treatment program, as more and more participants drop out. Therefore, there will be fewer participants providing data the further into the treatment you get. This can result in misleading low symptom scores toward the end of the program, as it could be the case that only the participants with the largest decrease in symptoms finish the program whilst the rest dropout. However, the dropout rate in this study was not higher than what is normally found in guided ICBT (Melville et al., 2010), and the statistical methods applied in this study (MLM) is known for handling missing data well. Fourth, since participants had to accept participating in the study it is likely that the sampling occurred in a non-random way and that sample selection bias was evident. It could be that those who participated believed more in their ability to carry out the treatment compared to those who did not participate in this study, something which could explain the high levels of treatment self-efficacy found in this study. Fifth, there are several methodological issues regarding the way that BGCSES assessing treatment self-efficacy was adapted to this study. The original scale has 20 items, but in the adapted version applied in this study the number of items is reduced to 15 items. The reduction of items was executed by other researchers before this study and the authors were not included in this process and thus have no knowledge regarding which items were withdrawn and why. It is also uncertain whether a pilot study has been carried out to test the instrument or not. These deviations from methodological rigor in the construction of the instrument measuring treatment self-efficacy may limit the validity of treatment self-efficacy in this study.

### Implications

The results from this study indicate that treatment self-efficacy for guided Internet-delivered therapy could be a predictor and moderator of symptom reduction and a predictor of dropout in PD and SAD. If these results are replicated in later studies, questionnaires measuring treatment self-efficacy could be implemented in guided Internet-delivered therapies so that this treatment can be offered to those who are most likely to benefit from it, and adaptions can be made to those patients who are less likely to benefit from it. Treatment self-efficacy could also be a useful way of identifying patients at risk of dropping out, in addition to being a valuable tool for working with specific areas of motivation to prevent dropout from guided Internet-delivered therapy. Treatment self-efficacy, as measured through the 15 items in the adapted version of the Bergen Genetic Counseling Self-Efficacy Scale, can easily be incorporated in a routine care setting as its administration is not time-consuming and demands little resources. This makes treatment self-efficacy a potential practical and implementation-friendly predictor and moderator in a routine care setting.

Although measuring the effectiveness of guided Internet-delivered therapy was not an aim in this study, the results are in line with previous research and confirming the effectiveness of guided Internet-delivered therapy (Andersson, 2018).

### Future Possible Directions

As pointed out in this paper, the results regarding predictors and moderators of dropout and successful treatment in Internet-delivered interventions is relatively scarce and inconsistent. An obvious reason is that there has not been conducted enough research. Another probable reason for the inconsistency in the literature pointed out by Beatty and Binnion (2016) is the great variation in study methodologies and procedures between studies on Internet-delivered interventions. Such variations complicate the interpretation of results, and examples are variations in length, content, the targeting of different mental disorders, sample size, statistical and analytic approaches and whether it is measuring efficacy or effectiveness. The lack of standardization and concise terminology within the field of Internet-delivered interventions has also been pointed out by others (Barak et al., 2009; Andersson, 2018; Smoktunowicz et al., 2020). Several researchers have presented possible solutions to the issue of inconsistent terminology in the field, and recommendations regarding statistical and analytical procedures (Hesser, 2015; Smoktunowicz et al., 2020). Increased awareness and consensus regarding the research process and language use, and the execution of these, could possibly facilitate the discovery of predictors and moderators in the field of Internet-delivered interventions and make such interventions more effective.

Future research on predictors and moderators should also investigate how adaptions can be made for the patients who are less likely to benefit from guided Internet-delivered therapy to increase their chances for experiencing positive treatment outcomes. This has not been the focus of many studies, which is a natural consequence of the literature being characterized by divergent and inconsistent results with few discovered robust predictors or moderators of treatment. It is argued that an essential step in this line of research would be to explore the possible reasons for dropout with participants in guided Internet-delivered therapy. This information would provide valuable insights into why dropout occurs and how it can be prevented through adaptations.

There are many unanswered questions regarding moderators of treatment outcome. A related and less researched field is the mechanisms through which guided Interned-delivered therapy operates, namely the mediators of change. There is little knowledge about how the specific components of guided Internet-delivered therapy are bringing about the effect on treatment outcome. The discovery of mediators in guided Internet-delivered therapy would contribute to this literature, making it possible to point out the specific components that makes the therapy effective. Furthermore, these components could be enhanced and adapted based on which components are deemed effective. Research on mediators of change usually require more frequent measurements of both process variables and outcome variables, and Internet-delivered interventions are well-suited for this as measures easily can be implemented into the programs without demanding time from a session or a therapist (Andersson, 2018). Due to the substantial share of patients not experiencing symptom reductions in guided Internet-delivered therapy, future research should aim to discover not only moderators, but also mediators of change in guided Internet-delivered therapy as this knowledge would contribute to making guided Internet-delivered therapy an effective treatment option for as many patients as possible.

## Conclusions

The results indicate that treatment self-efficacy in guided Internet-delivered therapy for anxiety disorders could be a predictor and moderator of symptom reduction, and a predictor of dropout. The results suggest that measuring treatment self-efficacy may be a valuable tool to identify patients at risk of dropping out. However, more research on treatment self-efficacy as a potential predictor and moderator of treatment outcome in guided Internet-delivered therapy is necessary as these results neither has been reported nor examined earlier.

## Data Availability Statement

The raw data supporting the conclusions of this article will be made available by the authors, without undue reservation.

## Author Contributions

AS was responsible for the execution of analyses and drafting the manuscript and contributed to the interpretation of results. TN contributed considerably to the manuscript as the head of eMeistring was responsible for study design and data collection and contributed considerably to the interpretation and drafting the manuscript. All authors have read and approved the final version of the publication.

## Funding

INTROMAT (INTROducing Mental health through Adaptive Technology). Award number: 259293.

## Conflict of Interest

The authors declare that the research was conducted in the absence of any commercial or financial relationships that could be construed as a potential conflict of interest.

## Publisher's Note

All claims expressed in this article are solely those of the authors and do not necessarily represent those of their affiliated organizations, or those of the publisher, the editors and the reviewers. Any product that may be evaluated in this article, or claim that may be made by its manufacturer, is not guaranteed or endorsed by the publisher.
