# Supplementary material for: Predicting Treatment Outcomes in Guided Internet-Delivered Therapy for Anxiety Disorders—The Role of Treatment Self-Efficacy
Source: Front Psychol. 2021 Oct 21;12:712421. doi: 10.3389/fpsyg.2021.712421 (PMC8566333; doi:10.3389/fpsyg.2021.712421)
Supplement: Supplementary file 1 [file Table_1.DOCX]

# Supplementary material

## A Treatment Self-Efficacy Questionnaire

Please indicate how confident you are that you will be able to cope with problems that may arise before, during and after use of the self-help program. Indicate your degree of confidence for each question on scale from 1 to 10:

| Can certainly not cope | | |  |  |  |  |  | Can certainly cope | |
| --- | --- | --- | --- | --- | --- | --- | --- | --- | --- |
| 1 | 2 | 3 | 4 | 5 | 6 | 7 | 8 | 9 | 10 |

|  | 1 | 2 | 3 | 4 | 5 | 6 | 7 | 8 | 9 | 10 |
| --- | --- | --- | --- | --- | --- | --- | --- | --- | --- | --- |
| Understand the text in the treatment program |  |  |  |  |  |  |  |  |  |  |
|  |  |  |  |  |  |  |  |  |  |  |
| Use computer and Internet |  |  |  |  |  |  |  |  |  |  |
|  |  |  |  |  |  |  |  |  |  |  |
| Ask the therapist in the weekly phone sessions regarding things that I do not understand. |  |  |  |  |  |  |  |  |  |  |
|  |  |  |  |  |  |  |  |  |  |  |
| Set aside sufficient time to carry out the exercises described in the treatment program. |  |  |  |  |  |  |  |  |  |  |
|  |  |  |  |  |  |  |  |  |  |  |
| Seek support from family and friends when I need it. |  |  |  |  |  |  |  |  |  |  |
|  |  |  |  |  |  |  |  |  |  |  |
| Set aside sufficient time to read and learn the content in the treatment program. |  |  |  |  |  |  |  |  |  |  |
|  |  |  |  |  |  |  |  |  |  |  |
| Stay motivated if the treatment program does not have immediate effect. |  |  |  |  |  |  |  |  |  |  |
|  |  |  |  |  |  |  |  |  |  |  |
| Staying in the treatment program despite a relapse (e.g., experiencing anxiety in a situation that I previously mastered). |  |  |  |  |  |  |  |  |  |  |
|  |  |  |  |  |  |  |  |  |  |  |
| Do exercises that I dread. |  |  |  |  |  |  |  |  |  |  |
|  |  |  |  |  |  |  |  |  |  |  |
| Staying in the treatment program despite a period of increased symptoms. |  |  |  |  |  |  |  |  |  |  |
|  |  |  |  |  |  |  |  |  |  |  |
| Ask for help from friends and family to complete exercises that I dread to do. |  |  |  |  |  |  |  |  |  |  |
|  |  |  |  |  |  |  |  |  |  |  |
| Work with the treatment program even though I have a busy everyday life. |  |  |  |  |  |  |  |  |  |  |
|  |  |  |  |  |  |  |  |  |  |  |
| Limit other tasks in everyday life so that I can work sufficiently with the self-help program. |  |  |  |  |  |  |  |  |  |  |
|  |  |  |  |  |  |  |  |  |  |  |
| Ask the therapist in the weekly phone sessions about difficulties with the exercises. |  |  |  |  |  |  |  |  |  |  |
|  |  |  |  |  |  |  |  |  |  |  |
| Ask friends, family, or others to help me with the exercises I will do. |  |  |  |  |  |  |  |  |  |  |

## B Structure Matrix of the Four Subscales of Self-Efficacy

|  | | | | |
| --- | --- | --- | --- | --- |
|  | Component | | | |
|  | 1 | 2 | 3 | 4 |
| SE_1 | ,322 | ,166 | ,792 | ,383 |
| SE_2 | ,124 | ,065 | ,714 | ,229 |
| SE_3 | ,455 | ,386 | ,788 | ,244 |
| SE_4 | ,919 | ,347 | ,303 | ,464 |
| SE_5 | ,401 | ,920 | ,231 | ,338 |
| SE_6 | ,911 | ,348 | ,323 | ,476 |
| SE_7 | ,678 | ,417 | ,356 | ,764 |
| SE_8 | ,549 | ,373 | ,333 | ,886 |
| SE_9 | ,468 | ,417 | ,318 | ,847 |
| SE_10 | ,492 | ,347 | ,343 | ,911 |
| SE_11 | ,354 | ,948 | ,178 | ,417 |
| SE_12 | ,864 | ,372 | ,328 | ,586 |
| SE_13 | ,861 | ,425 | ,307 | ,518 |
| SE_14 | ,616 | ,455 | ,670 | ,365 |
| SE_15 | ,376 | ,966 | ,216 | ,342 |

| Extraction Method: Principal Component Analysis.  Rotation Method: Oblimin with Kaiser Normalization. |
| --- |
